# Supplementary material for: Accurately clustering biological sequences in linear time by relatedness sorting
Source: Nat Commun. 2024 Apr 8;15:3047. doi: 10.1038/s41467-024-47371-9 (PMC11001989; doi:10.1038/s41467-024-47371-9)
Supplement: Supplementary file 1 — Supplementary Information [file 41467_2024_47371_MOESM1_ESM.pdf]

Supplementary Information for

Accurately clustering biological sequences in linear time by relatedness sorting

By Erik S. Wright

TABLE OF CONTENTS

Supplementary Figure 1. Selecting default values for user-specified parameters..........................2

Supplementary Figure 2. Cluster origins on 411 large TIGRFAM protein families..........................3

Supplementary Figure 3. Benchmarking on 3,001 small TIGRFAM protein families. 4

Supplementary Figure 4. Differing efficiency with the use of multiple processors.........................5

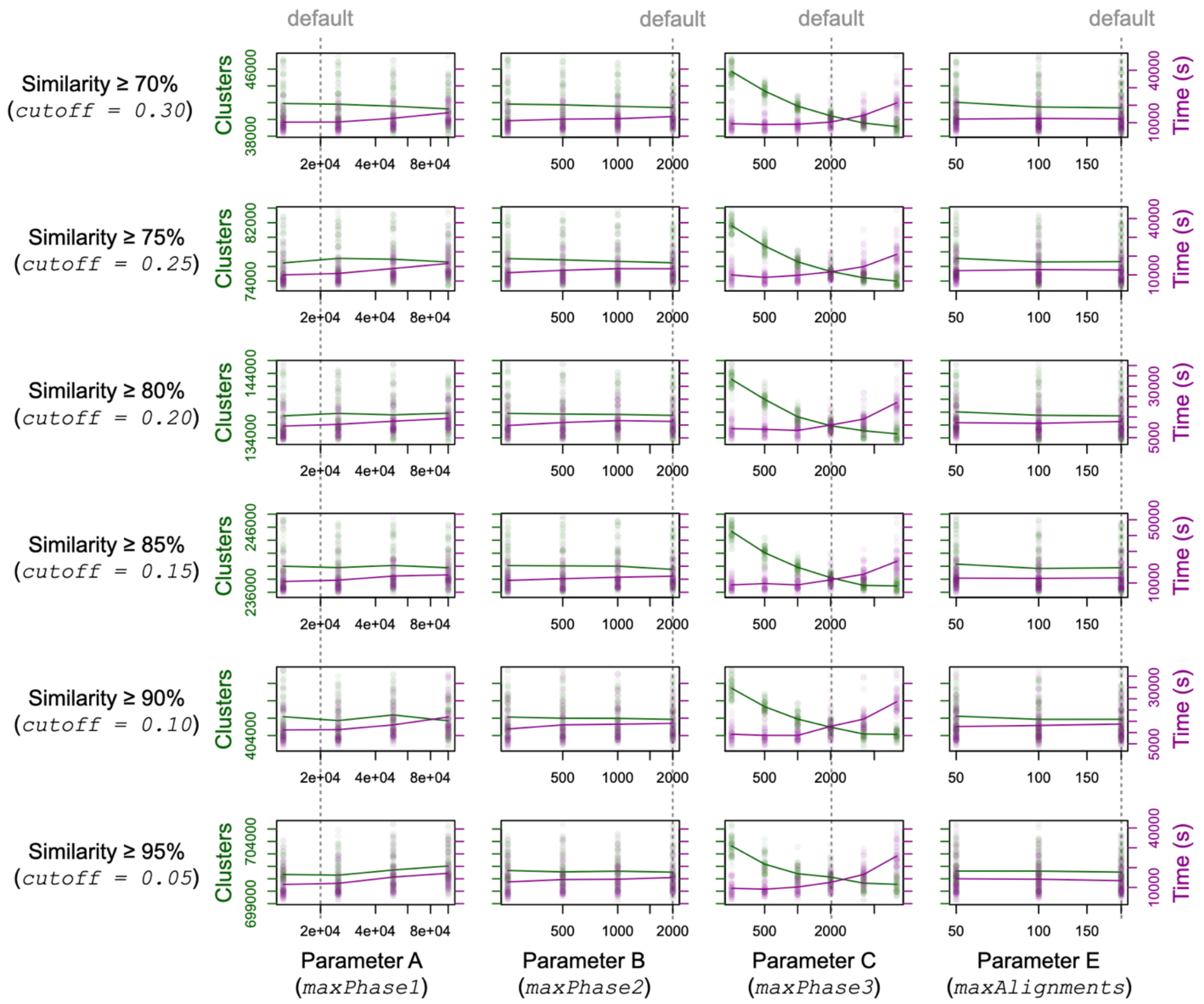

Supplementary Figure 1. Selecting default values for user-specified parameters. Each point represents the number of clusters (green) or elapsed time (purple) for a unique combination of values for parameters *A*, *B*, *C*, and *E*. Results are stratified by parameter (column) and similarity threshold (row). Mean values are connected by lines. The clustering input consisted of 1,807,555 non-coding RNA sequences with fewer than 800 nucleotides from the RNAcentral database (*rnacentral.org*). Corresponding parameter names in Clusterize are shown in *italics*. All x-axes are shown on a log-scale and y-axes on a linear scale. Default parameter values (gray vertical dashed lines) were selected to balance minimizing the number of clusters and the elapsed time. Note that this analysis was conducted with distributed computing, and elapsed times may differ across computers.

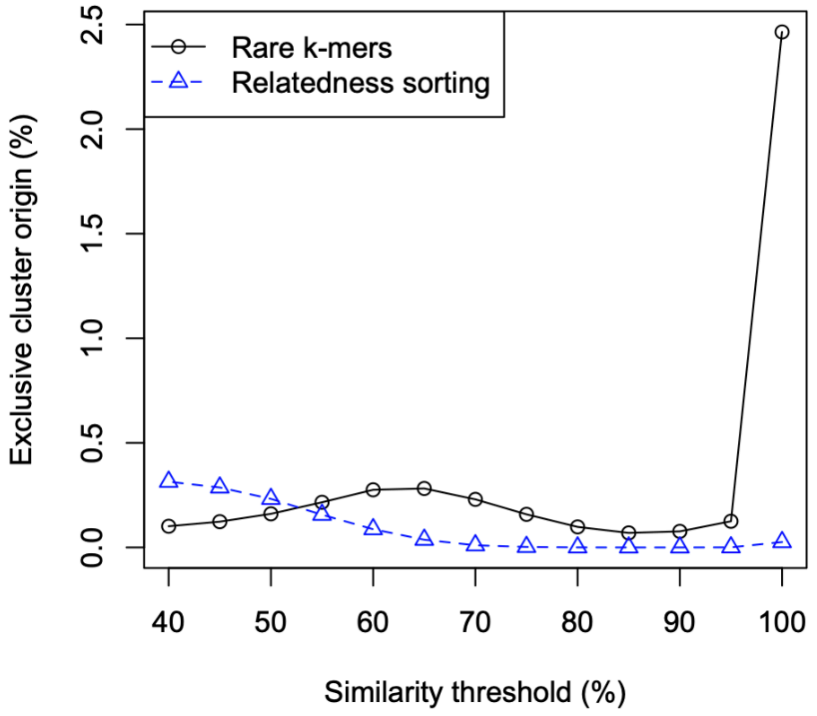

Supplementary Figure 2. Cluster origins on 411 large TIGRFAM protein families. The average percentage of clustered sequences (y-axis) originating exclusively from each method is shown as a function of the user-specified similarity threshold (x-axis). More clustered sequences originated exclusively from rare k-mers at high similarity thresholds and relatedness sorting at low similarity thresholds.

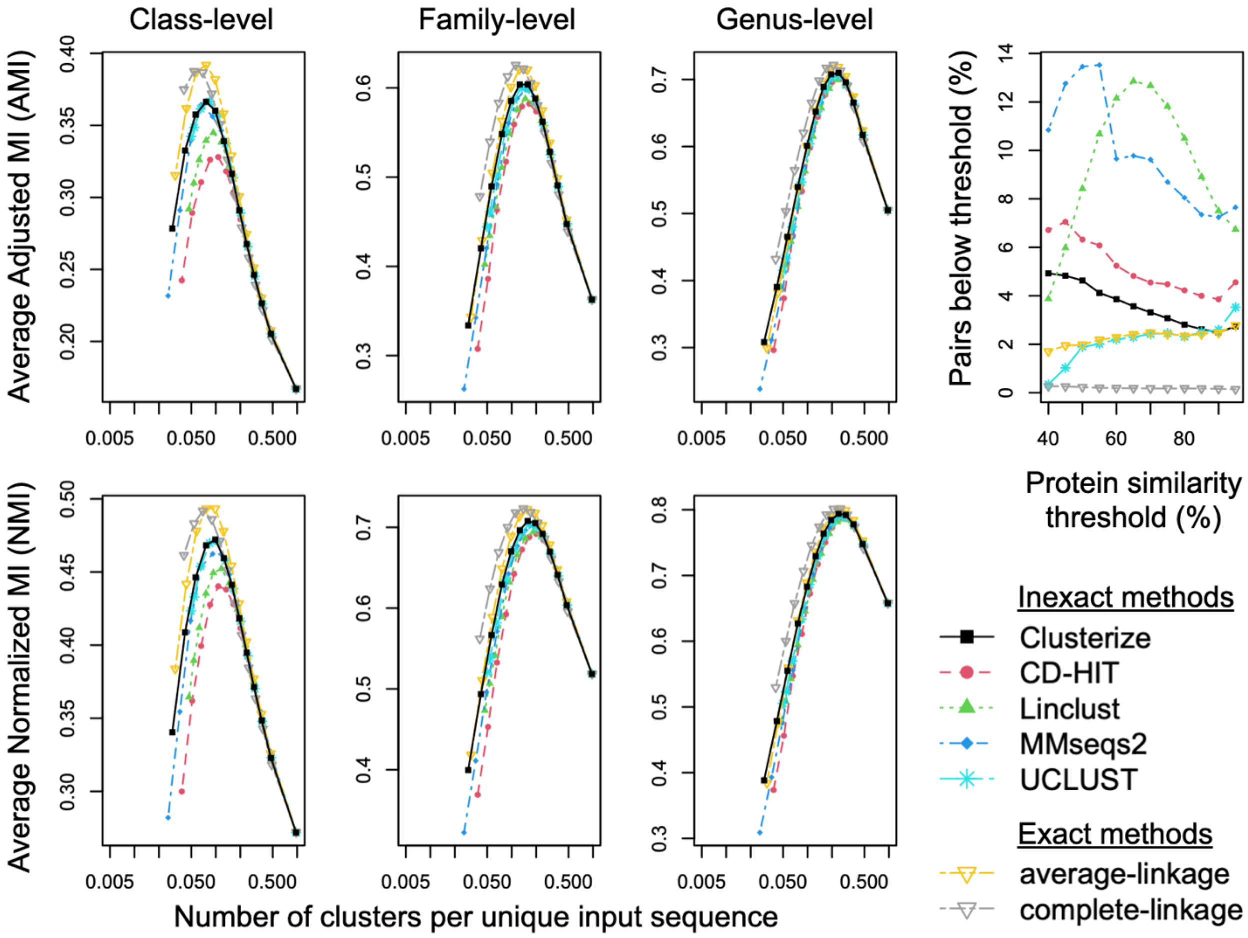

Supplementary Figure 3. Benchmarking on 3,001 small TIGRFAM protein families. Inexact clustering programs were ranked similarly on small TIGRFAM protein families as on large TIGRFAM protein families (Fig. 2), with Clusterize among the top three programs. Both exact clustering methods, average-linkage and complete-linkage, outperformed all inexact clustering programs.

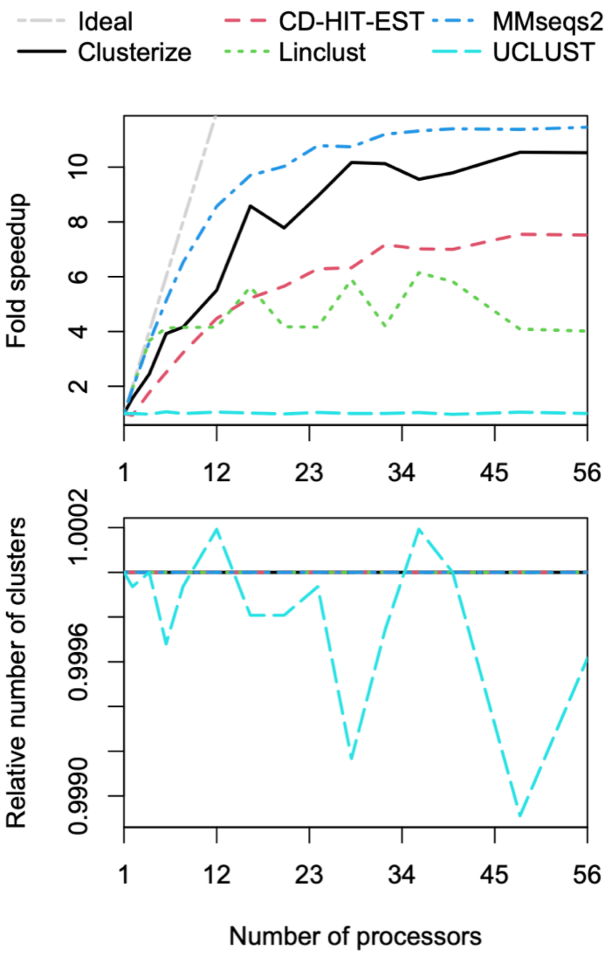

Supplementary Figure 4. Differing efficiency with the use of multiple processors. SARS-CoV-2 genomes were clustered using different numbers of processors (top). The number of input sequences was selected for each program such that it required less than about 10 minutes to cluster the sequences using 8 processors. All programs except UCLUST achieved a modest speedup using more than one processor. The fold-difference in number of returned clusters is shown relative to using a single processor (bottom). UCLUST returned different results when specifying a number of threads, although the results were reproducible for the same number of threads. All other programs returned the same number of clusters across processors, as shown by the overlapping lines.
