# Supplementary material for: Accurately clustering biological sequences in linear time by relatedness sorting
Source: Nat Commun. 2024 Apr 8;15:3047. doi: 10.1038/s41467-024-47371-9 (PMC11001989; doi:10.1038/s41467-024-47371-9)
Supplement: Supplementary file 3 — Reporting Summary [file 41467_2024_47371_MOESM3_ESM.pdf]

nature portfolio

Corresponding author(s): Erik S. Wright

Last updated by author(s): 03/14/2024

Reporting Summary

Nature Portfolio wishes to improve the reproducibility of the work that we publish. This form provides structure for consistency and transparency in reporting. For further information on Nature Portfolio policies, see our Editorial Policies and the Editorial Policy Checklist.

Statistics

For all statistical analyses, confirm that the following items are present in the figure legend, table legend, main text, or Methods section.

n/a	Confirmed	
☐	☒	The exact sample size (*n*) for each experimental group/condition, given as a discrete number and unit of measurement
☐	☒	A statement on whether measurements were taken from distinct samples or whether the same sample was measured repeatedly
☒	☐	The statistical test(s) used AND whether they are one- or two-sided<br>*Only common tests should be described solely by name; describe more complex techniques in the Methods section.*
☒	☐	A description of all covariates tested
☒	☐	A description of any assumptions or corrections, such as tests of normality and adjustment for multiple comparisons
☐	☒	A full description of the statistical parameters including central tendency (e.g. means) or other basic estimates (e.g. regression coefficient) AND variation (e.g. standard deviation) or associated estimates of uncertainty (e.g. confidence intervals)
☒	☐	For null hypothesis testing, the test statistic (e.g. *F*, *t*, *r*) with confidence intervals, effect sizes, degrees of freedom and *P* value noted<br>*Give P values as exact values whenever suitable.*
☒	☐	For Bayesian analysis, information on the choice of priors and Markov chain Monte Carlo settings
☒	☐	For hierarchical and complex designs, identification of the appropriate level for tests and full reporting of outcomes
☒	☐	Estimates of effect sizes (e.g. Cohen's *d*, Pearson's *r*), indicating how they were calculated

Our web collection on statistics for biologists contains articles on many of the points above.

Software and code

Policy information about availability of computer code

Data collection	Clusterize is part of the DECIPHER (v2.30) package for the R programming language available from Bioconductor (DOI:10.18129/B9.bioc.DECIPHER).
Data analysis	See Materials and Methods section for a description of all programs used in this study, along with their settings and version numbers.

For manuscripts utilizing custom algorithms or software that are central to the research but not yet described in published literature, software must be made available to editors and reviewers. We strongly encourage code deposition in a community repository (e.g. GitHub). See the Nature Portfolio guidelines for submitting code & software for further information.

Data

Policy information about availability of data

All manuscripts must include a data availability statement. This statement should provide the following information, where applicable:

- Accession codes, unique identifiers, or web links for publicly available datasets
- A description of any restrictions on data availability
- For clinical datasets or third party data, please ensure that the statement adheres to our policy

TIGRFAM families are available from Zenodo (https://doi.org/10.5281/zenodo.10019584) in compressed FASTA format. Human Microbiome Project data was downloaded from the HMP Portal (https://portal.hmpdacc.org/). SARS-CoV-2 sequences were downloaded from GISAID (https://gisaid.org) on September 16th,

2022, and GISAID explicitly forbids redistribution without authorization. Sequences extracted from bacterial genomes are available from Zenodo (https://doi.org/10.5281/zenodo.10030000 and https://doi.org/10.5281/zenodo.10031801).

Human research participants

Policy information about studies involving human research participants and Sex and Gender in Research.

Reporting on sex and gender	N/A
Population characteristics	N/A
Recruitment	N/A
Ethics oversight	N/A

Note that full information on the approval of the study protocol must also be provided in the manuscript.

Field-specific reporting

Please select the one below that is the best fit for your research. If you are not sure, read the appropriate sections before making your selection.

☒ Life sciences ☐ Behavioural & social sciences ☐ Ecological, evolutionary & environmental sciences

For a reference copy of the document with all sections, see nature.com/documents/nr-reporting-summary-flat.pdf

Life sciences study design

All studies must disclose on these points even when the disclosure is negative.

Sample size	All 411 large plus 3,001 TIGRFAM families. All Human Microbiome Project and all SARS-CoV-2 sequences were included that met the constraints described in the Materials and Methods. All protein and protein coding sequences with protein functional annotations were included.
Data exclusions	None.
Replication	Each TIGRFAM family was tested independently (n = 411 or 3,001). All other sequences were tested at multiple input sizes (see Methods).
Randomization	All tested sequences were randomly ordered by sequence or genome in the input, as to mitigate any potential issues with input sequence ordering.
Blinding	N/A

Reporting for specific materials, systems and methods

We require information from authors about some types of materials, experimental systems and methods used in many studies. Here, indicate whether each material, system or method listed is relevant to your study. If you are not sure if a list item applies to your research, read the appropriate section before selecting a response.

Materials & experimental systems

n/a	Involved in the study	
☒	☐	Antibodies
☒	☐	Eukaryotic cell lines
☒	☐	Palaeontology and archaeology
☒	☐	Animals and other organisms
☒	☐	Clinical data
☒	☐	Dual use research of concern

Methods

n/a	Involved in the study	
☒	☐	ChIP-seq
☒	☐	Flow cytometry
☒	☐	MRI-based neuroimaging
